# Advancing the Diagnosis of Diabetic Neuropathies: Electrodiagnostic and Skin Autofluorescence Methods

**DOI:** 10.3390/jpm14080884

**Published:** 2024-08-21

**Authors:** Dan Trofin, Bianca-Margareta Salmen, Teodor Salmen, Daniela Marilena Trofin, Delia Reurean-Pintilei

**Affiliations:** 1 Department of Diabetes, Nutrition and Metabolic Diseases, Consultmed Medical Centre, 700544 Iasi, Romania; trofin.dan@umfiasi.ro (D.T.); delia.pintilei@usm.ro (D.R.-P.); 2 Department of Biomedical Sciences, Faculty of Medical Bioengineering, University of Medicine and Pharmacy “Grigore T. Popa” Iasi, 700454 Iasi, Romania; 3 Doctoral School of Carol Davila, University of Medicine and Pharmacy, 020021 Bucharest, Romania; bianca-margareta.mihai@drd.umfcd.ro; 4 Neurology Clinic, The Rehabilitation Hospital, 700661 Iasi, Romania; dr.danielatrofin@gmail.com; 5 Department of Medical-Surgical and Complementary Sciences, Faculty of Medicine and Biological Sciences, “Ștefan cel Mare” University, 720229 Suceava, Romania

**Keywords:** electroneurography, entrapment neuropathy, AGEs, skin autofluorescence, HbA1c

## Abstract

Introduction: Diabetic neuropathy (DN) is a generic term for various neuropathies coexisting in a single patient. Clinical diagnosis alone can be misleading, yet routine electrodiagnostic studies in diabetes care are rare. Skin autofluorescence (SAF) is a recognized DN risk factor with potential screening value. This article highlights the diagnostic challenges and raises awareness of the often underdiagnosed neuropathic conditions in diabetes patients. Material and Methods: We present common entrapment neuropathy cases from our diabetes clinic’s electrodiagnosis laboratory in Iași, Romania. We selected seven type 2 diabetes patients with sensory or sensory-motor distal polyneuropathy and atypical DN presentations investigated through electroneurography (ENG) and electromyography (EMG) with the Neurosoft^®^ EMG instrument and SAF measured by standard procedures. Subsequently, a narrative literature review was conducted. Results: Entrapment neuropathies were diagnosed in all the patients: three carpal tunnel syndromes, two ulnar neuropathies (one proximal, one distal), one peroneal neuropathy, and one case of meralgia paresthetica. The lower-limb cases showed radiculoplexopathy, and there was one case of superficial radial nerve neuropathy. The SAF values ranged from 2.5 AU to 3.4 AU. Conclusions: Electrodiagnosis is essential for detecting focal neuropathies in patients with sensory-motor distal polyneuropathy. Elevated SAF levels may correlate with symptom severity, although further research, including large cohorts, is needed.

## 1. Introduction

Although “Diabetic Neuropathy” (DN) as a term has various definitions and classifications, it is considered general nomenclature encompassing multiple neuropathies, as none are strictly unique to diabetes mellitus (DM) [1]. In addition to mononeuropathies (such as carpal tunnel syndrome (CTS), cubital nerve neuropathy, peroneal neuropathy (PN), and cranial nerve III, IV, VI, or VII involvement), DN can frequently present as sensory, sensory-motor, or autonomic polyneuropathies, as well as radiculopathies and plexopathies in various forms [1,2]. The best example of a true polyradiculoneuropathy is DN itself, which is typically straightforward to diagnose clinically. However, since the symptoms and signs can manifest both proximally and distally, electrodiagnosis (EDX) emerges as a necessity in challenging differential diagnoses (DDx) [1,3]. For example, electrophysiologic data support the diagnosis of widespread, persistent sensorimotor peripheral neuropathy. Additionally, EDX studies may reveal a superimposed, subacute denervation process [1,3]. A careful investigation protocol involving both EMG and magnetic resonance imaging (MRI) would clarify upon the opportunity of surgery in such a situation [4].

The common presentations of neuropathies include a range of symptoms, such as pain and discomfort along the anatomical tract of one or more peripheral nerves, more pronounced at rest or at night, and numbness in the distal limbs [5,6]. Additionally, cramps, muscle soreness, muscle loss, and other non-specific symptoms related to the autonomic or cranial nerves can occur [6,7]. Also, it can lead to muscle weakness and balance impairment, which can alter patients’ functioning. The alteration in patients’ functioning, along with chronic pain, can significantly affect their quality of life. Preventive measures and management after diagnosis are important for comprehensive care. One important preventive measure is tight glycemic control. Hypoglycemic medications and supplementation are two ways to obtain this [8]. Along with medication, rehabilitation interventions can be used to diminish chronic pain and improve functioning [9]. Rehabilitation interventions include patient education and activity modification to increase physical activity, as well as physiotherapy techniques such as diathermy and low-level laser therapy [10,11]. DN symptoms are commonly seen in the daily practice of a multidisciplinary medical clinic that treats and diagnoses DM. Patients experiencing some or all of these symptoms would be referred for EDX studies. When reporting EDX results, it is important to consider clinical symptoms and referral diagnoses [1], as every investigation should establish a correlation between clinical impairment and electrophysiologic abnormalities [1,3].

Integrating electroneurography (ENG) to study nerve conductivity and electromyography (EMG) with needle electrodes to assess muscle function—either separately or combined—provides highly sensitive tests within electroneuromyography (ENMG) [12,13]. These tests enable clinicians to detect subtle, subclinical abnormalities that patients may not be aware of. For example, a patient with DM and polyneuropathy referred to the EMG laboratory may show electrophysiologic signs of underlying ulnar neuropathy (UlN), even without symptoms. While ENMG effectively evaluates electrophysiologic abnormalities, the results must always be interpreted in the context of a thorough anamnesis and clinical examination [1,3,14].

In the frequent case of DN-related numbness and tingling in both feet, in nerve conduction studies, a relative slowing of the median nerve may be recorded across the wrist, even though the patient has not reported any pain, paresthesia, or hand weakness. A clinical CTS is most likely improbable; instead, EDX indicates an underlying polyneuropathy of the median nerve from the wrist [3]. This differentiation is crucial as surgery is not warranted, underscoring the importance of a thorough anamnesis and clinical history, along with a physical examination, for the accurate performance and interpretation of nerve conduction and EMG evaluations [3,15,16].

DN can manifest in many different forms, coexisting with the classical distal symmetrical presentation: when affecting the lumbar plexus, diabetic amyotrophy (DA) is also referred to as proximal DN or plexopathy [3,4]. Patients with DM may also develop femoral neuropathy, most often as a result of nerve infarction, but, typically, this happens in conjunction with a more widespread polyradiculoplexopathy—DA; in other cases, patients may experience painful lumbosacral plexopathy [4]. Proximal DN involvement or diabetic mononeuritis multiplex, also known as diabetic polyradiculopathy or, simply, DA, describes the same illness. This list of terminology has been updated to include diabetes lumbosacral radiculoplexus neuropathy (DLSRPN) [3]. Traditionally, DA affects predisposed locations such as nerve roots and the upper lumbar plexus. Hence, radiculoplexopathy is the true diagnosis for DA and the fundamental cause is microscopic vasculitis that results in nerve ischemia [1,3].

DA usually manifests as unilateral disease. In some, within the first few weeks or months of the initial manifestation, the same process may impact the contralateral side. Although recovery is generally favorable, it can take several months or longer periods [3]. This context presents DDx challenges, especially in elderly patients or those with a long history of radicular problems. Nonetheless, these clinical and EDX considerations align with less common associations, such as ulnar nerve entrapment in the Guyon canal and meralgia paresthetica, as well as more common ones like tarsal tunnel syndrome (TTS) [17].

Patients with DM are prone to these neuropathic conditions due to their long-term elevated blood sugar levels, which alter multiple protein structures and functions. Glycation affects albumin, globulins, fibrinogen, and collagen, contributing to all chronic DM complications, including DN. The development of advanced glycation end products (AGEs) is one of the primary causes of these issues [18,19]. AGEs’ accumulation and synthesis are facilitated by the combined effects of obesity, elevated blood glucose, inflammation and oxidative stress. An increase in the creation of these products or a delay in their removal is also observed naturally as people age, but also in several chronic disorders such as renal disease and neurodegenerative pathologies. The impact of proteins and peptides varies with their lifespans; those with longer durations are associated with an accumulation of AGEs, resulting in specific complications [18,19]. Even before the onset of clinical neuropathic manifestations, skin autofluorescence (SAF) was demonstrated to be correlated with the severity of peripheral and autonomic nerve disorders associated with DM. Measuring skin AGEs through SAF could be a quick and easy clinical tool for assessing the likelihood of DM progressing to multisystemic complications [20].

The purpose of this article is to raise awareness of the focal complications that may occur in patients with DM. Moreover, to promote the utility of accessible and integrated EDX capabilities in terms of the ease of access, we also aim to highlight the value of complementary investigative techniques, particularly the study of skin autofluorescence for measuring advanced glycation end products. The combined use of SAF and EDX is not well documented in the scientific literature, making this an area for further exploration. By presenting a series of common entrapment neuropathies encountered in our clinic, we seek to underscore the importance of electrodiagnostic studies and the potential role of SAF as a screening tool in managing DN.

## 2. Materials and Methods

We retrospectively present an intervention report on a series of 7 EDX cases of patients with DM, who were referred for atypical signs of neuropathic involvement to the EDX laboratory of a single interdisciplinary medical center specialized in the diagnosis and treatment of DM, serving the entire eastern region of Romania. The patients were investigated using SAF and ENMG, with their HbA1c levels routinely checked. Both nerve conduction studies and needle EMG investigations were conducted using a two-channel Neurosoft^®^ EMG instrument. The nerve conduction studies were performed based on the values established in our laboratory, according to the device’s technical settings (Table 1 and Table 2). The method is based on applying an electrical impulse to specific sites along a peripheral nerve and recording the generated action potential.

The motor nerve conduction study assesses the compound muscle action potential (CMAP), which is the motor response of the muscle collected by surface electrodes as a result of stimulating the motor nerve fibers that provide innervation. We analyze various parameters, such as the latency (or motor conduction time), which is the interval between the electrical stimulation and the onset of the action potential (CMAP). In the case of a focal neuropathy, it is evident that the conduction time will be prolonged; the method therefore allows us to accurately identify the entrapment site responsible for the conduction block (several anatomical regions are typically prone to entrapment). To achieve this, we analyze parameters such as the CMAP amplitude or the motor conduction velocity (reported to the segment of the nerve situated between two stimulation sites: a distal and a proximal).

The EMG device also provides the means to measure late responses such as the F wave, which is the depolarization of the cell situated in the anterior horn of the spine as a response to motor fibers’ electrical stimulation. When comparing the values recorded in the two limbs, the proximal segment of the nerve is being assessed, hence showing the utility in both radiculopathies and neuropathies.

Sensory studies were performed antidromically, with the sensory nerve action potential (SNAP) amplitudes measured from baseline to the negative peak of the potential. We assessed similar parameters as in the motor conduction protocol (amplitude of the SNAP, sensitive nerve conduction velocity, eventual increase in latency along a suspected entrapment site, or peak to peak amplitude suggesting the number of activated fibers, or the rise-time). In entrapment lesions, the practical value of the sensitive parameters tends to be higher than the motor one (especially in mild to moderate neuropathies).

Needle EMG is used to record and measure the motor unit action potential (MUAP), the activity of the region of the motor unit situated nearby to the recording area of the needle electrode (0.5 mm). We used concentric needle electrodes, which record MUAPs with 2–15 msec duration, 200 µV–3 mV, bi-, tri- or polyphasic configuration. Usually, in chronic neuropathies, the neurogenic pattern is suggested by the complex morphology of the MUAPs along a decreased recruitment. The needle EMG evaluation is essential to differentiate between myopathic and neurogenic patterns. All the patients were informed and provided written consent in accordance with ethical principles. The participants were all above 18 years old, with a history of type 2 DM (T2DM) and a clinical diagnosis of DN. There was no standardization of T2DM treatment. The patients did not have pacemakers and were not on anticoagulant treatment, although these are not absolute contraindications if specific safety precautions are followed. The glycemic and HbA1c levels were monitored according to the guidelines [21].

The SAF was assessed on the occasion of presentation for EDX by using the AGE Reader™ (DiagnOptics Technologies, Groningen, The Netherlands). This method is noninvasive and uses the autofluorescent properties of some advanced glycation end products, expressing the number of accumulated AGEs in arbitrary units (AUs). A specific wavelength of light is emitted by the device onto the skin, typically on the forearm of the patient. This light causes the AGEs present in the skin to fluoresce, or emit light, which is then detected and quantified by the device. The level of fluorescence correlates with the concentration of AGEs in the tissue. This method provides a quick and painless, and also reliable, evaluation of long-term metabolic stress as well as glycation, which can be indicative of an increased risk of DM complications. SAF has gained recognition as a potential screening tool in clinical practice, offering insights into a patient’s long-term metabolic health beyond more traditional blood glucose biomarkers, such as hemoglobin A1c [22,23,24]. Cut-off values have been proposed both for individuals diagnosed with DM and for the general population; however, customizing these thresholds according to diverse demographic groups remains an unmet need. The technical and methodological specifics have been detailed in prior publications [25,26].

### Ethical Aspects of the Research

The investigation adhered to the principles outlined in the Declaration of Helsinki and received approval from the Institutional Ethics Committee of Consultmed Hospital in Iași County, Romania (protocol number CMD102018014, dated 29 May 2024). The objectives and methods of this study were explained to the patients, who consented to the utilization and publication of the data collected, and informed consent was obtained.

## 3. Results

### 3.1. Clinical Case 1

A 58-year-old male patient with a long history of DM (over 10 years), currently treated with metformin, long-acting insulin analog, glucagon-like peptide-1 receptor agonist (GLP-1 Ra), and sodium-glucose co-transporter 2 inhibitors (SGLT2i). He has a history of bilateral surgically treated CTS (right in 2022, left in 2019) and disc herniation at the L5-S1 level with bilateral S1 radicular syndrome, experiencing progressive paresthesia and distal dysesthesia of the limbs.

**Figure 1:**
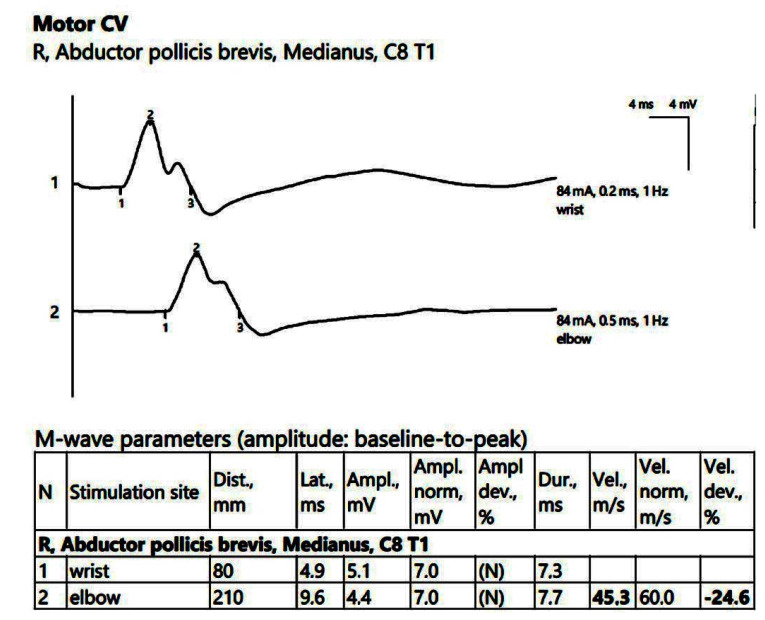
Patient 1. Motor conduction parameters in the right hand (the negative (upwards) action potentials generated by distal, above wrist (1) and proximal, at the elbow (2) stimulation with the active electrode situated on the *abductor pollicis brevis muscle,* allow us to divide the distance between the 2 stimulation sites (measured in mm) and the conduction time along that specific segment of the investigated nerve (proximal latency—distal latency); hence, the conduction velocity decreased in our patient, 45.3 m/s, suggesting the decrese in conduction velocity from the wrist towards the palm, whereas the conduction between the elbow and the wrist remains stationary). Note—the images are generated with Neurosoft device for EMG.

ENG showed a low compound muscle action potential (CMAP) amplitude on the right peroneal nerve (within reference values) without a decrease in the motor nerve conduction velocity (MCV). Additionally, low sensory nerve conduction velocity (SCV) was recorded symmetrically in the superficial peroneal and sural nerves bilaterally. In the upper limbs, there was reduced CMAP amplitude on the right median nerve, with temporal dispersion phenomena and slowing of the MCV (Figure 1).

**Figure 2:**
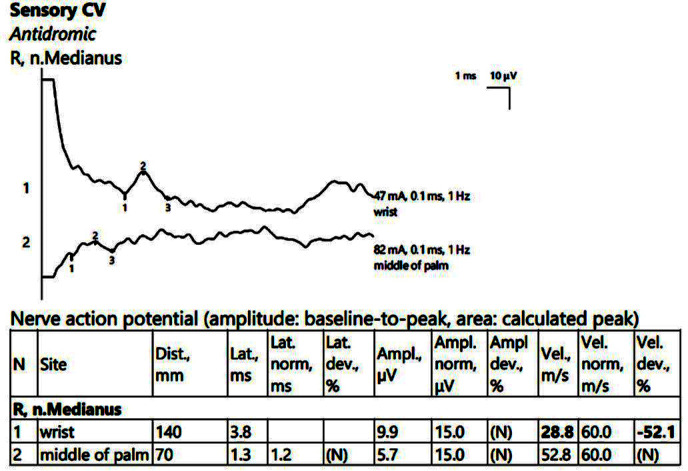
Patient 1. Stimulation performed above wrist and in mid-palm, with active electrode located on the index finger. Sensory conduction parameters in this right hand (low SNAP amplitudes (both because of the conduction block (first SNAP, 1) and the generalized polyneuropathy (the second SNAP, obtained by stimulation in the middle of the palm, 2, which should have been within normal values if the patient did not have DM), and decreased velocity across the carpal tunnel also suggested by a delayed latency between the two points). The conduction velocity is only 28.8 m/s, along with increased distal latency, 3.8 ms and lower SNAP amplitude (almost 50% compared to the wrist value) Note—the images are generated with Neurosoft device for EMG.

A low SCV when passing through the right carpal tunnel (Figure 2) was observed. The MCV ranged within normal limits on the left, with the SCV slightly decreased on the left. There were sensitive parameters within normal limits on the bilateral ulnar nerve.

In this case, the EDX exam is suggestive of a complex clinical polyneuropathic pattern: bilateral CTS (moderate on the right) and distal DN. Mild mixed lumbosacral radiculoplexopathy may be associated.

### 3.2. Clinical Case 2

A 57-year-old female patient with an 8-year history of T2DM, treated with metformin, SGLT2i, and dipeptidyl-peptidase 4 inhibitor, with a history of a panaritium on the third finger of her left hand, complicated by osteitis that required distal phalanx amputation in July 2022. She presented with chronic symptoms of distal paresthesia and dysesthesia of the limbs, and bilateral hand pain. She also exhibited amyotrophy of the thenar regions bilaterally, but without involvement of the first interosseous space, motor deficits (proximal or distal), or fasciculations.

**Figure 3:**
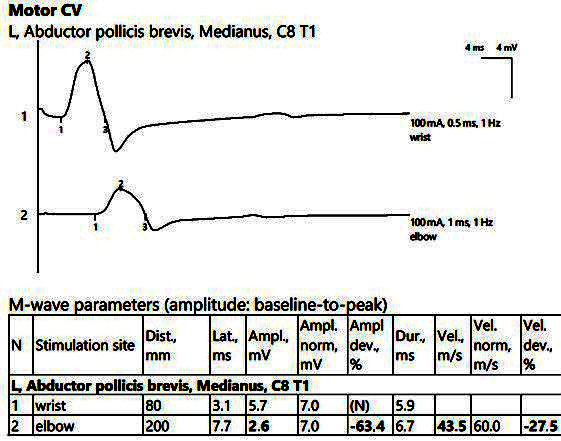
Patient 2. Motor conduction parameters in the left hand, with active electrode placed on the *abductor pollicis brevis* muscle’s belly (important decrease in the amplitude of the CMAP at the proximal stimulation (2, at the elbow) compared to the distal stimulation (1, above the wrist), visible when stimulating at the elbow, 2), >50%, with motor conduction velocity of only 43.5 m/s. Note—the images are generated with Neurosoft device for EMG.

ENMG showed reduced CMAP amplitudes of the median nerve bilaterally from the proximal stimulations, with increased temporal dispersion phenomena, increased distal latencies, and bilateral MCV slowing, as seen in Figure 3.

**Figure 4:**
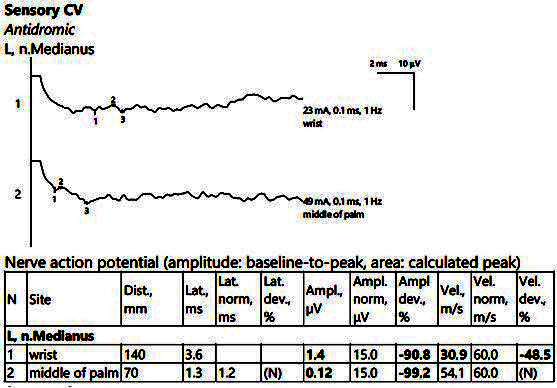
Patient 2. Sensory conduction parameters in the left hand (low amplitudes of the SNAP with a decrease in the velocity across the tunnel (30.9 m/s), visible when stimulating above the wrist (1) as compared to mid-palm stimulation (2)). Both SNAP amplitudes are decresead here in context of the polyneuropathic condition. Note—the images are generated with Neurosoft device for EMG evaluation.

The electrophysiological parameters were within normal limits on the bilateral ulnar nerve (motor and sensitive). There were also low SCVs bilaterally on the median nerve at the passage through the carpal tunnel, with increased distal latencies and SNAP of low amplitudes, as shown in Figure 4.

**Figure 5:**
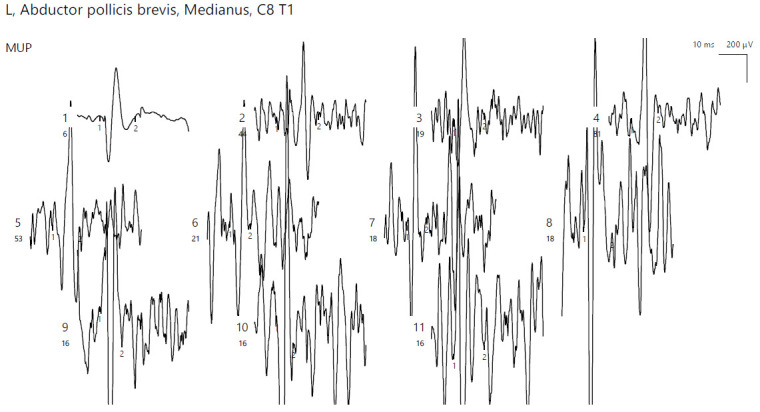
Patient 2. Neurogenic pattern recored with a concentric needle electrode in the left APB (increased amplitudes, polyphasic morphology; the absence of spontaneous pathologic activity adds up to the chronicity of the described condition).

EMG of the abductor pollicis brevis (APB) bilaterally showed no pathological spontaneous activity. A neurogenic pattern was present, with the motor unit action potentials (MUAPs) displaying large amplitudes, polyphasia, and increased duration (Figure 5). The left deltoid showed a normal pattern.

In the lower limbs, there were reduced CMAP amplitudes of the peroneal nerve bilaterally, with low MCVs and increased distal latencies. Additionally, low SCVs were observed bilaterally on the sural and superficial peroneal nerves.

The results suggest a chronic bilateral CTS, along with distal DN in the lower limbs.

### 3.3. Clinical Case 3

A 32-year-old female with a 3-year history of DM, treated with metformin and clinically diagnosed with DN, presented with a 3-month history of progressively painful decreased motility, resulting in an immobile fifth finger on the left hand and paresthesia in the corresponding hypothenar eminence. During this period, she underwent 2 weeks of medical rehabilitation for clinical UlN and associated bilateral alternating cervical-brachialgias. MRI confirmed multi-level cervical disc protrusions with radicular contacts at C5–C6–C7, more pronounced on the right than the left. According to her history and clinical reports, she experienced mild improvement in motility and better pain relief after physical therapy (including transcutaneous electrical nerve stimulation (TENS), neuromuscular electrical stimulation (NMES), and proprioceptive neuromuscular facilitation (PNF)), up to 2 weeks before the EDX. She reported an occupational history of prolonged tight contact between the ulnar side of her forearm and the desk while working.

**Figure 6:**
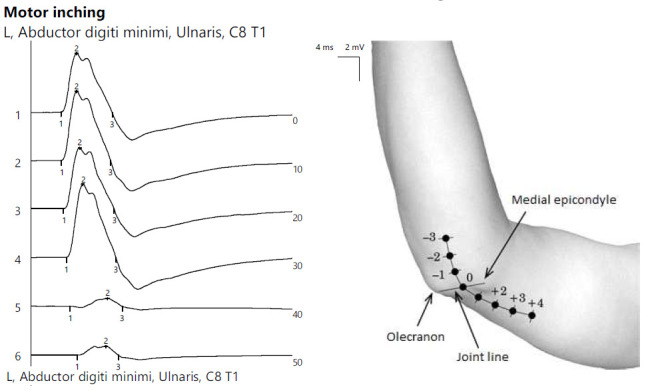
Patient 3. Focal ulnar neuropathy at the elbow (significant decrease in amplitudes of CMAPs when stimulating across the elbow, orientating the confirmation of the diagnosis).

ENG showed the reduced CMAP amplitude of the left ulnar nerve with a decrease upon proximal stimulation. Motor inching at the elbow revealed an abrupt drop in amplitude between the distal and proximal segments, indicating a focal lesion (Figure 6).

The MCV was within normal limits at this level. A decreased CMAP amplitude was measurable on the left radial nerve (compared to right) and a decreased SCV on the left ulnar nerve; otherwise, the findings were within normal limits in other territories. Along with the clinical history of DN, the EDX examination adds elements suggestive of a left UlN at the elbow and an associated cervical-brachial syndrome.

### 3.4. Clinical Case 4

A 48-year-old male with a 5-year history of DM, treated with metformin and clinically diagnosed with DN, presented with a history of difficulties in dorsiflexion of the right forefoot, with insidious onset and progressive worsening over the last month. Clinically, the MRC was 4/5 for right forefoot dorsiflexion. The patient has a history suggestive of practicing the “crossed legs” position and prolonged squatting while working. He also had a history of L4–L5 and L5–S1 disc protrusions with minimal radicular conflicts.

**Figure 7:**
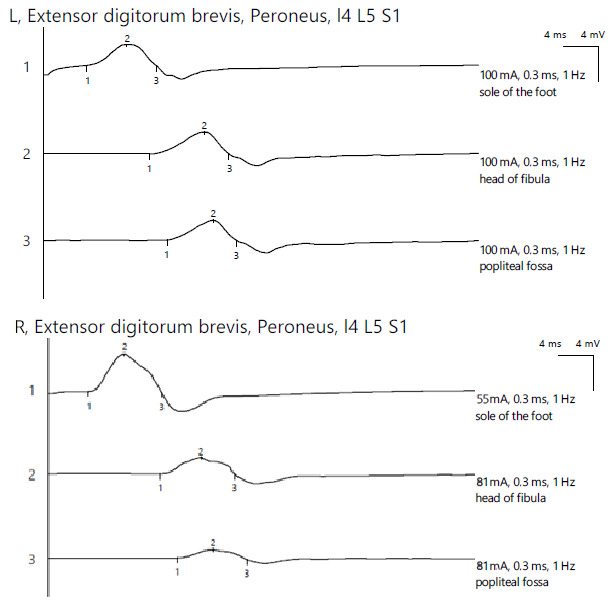
Patient 4. Comparative peroneal motor nerve conduction studies, with motor stimulation at ankle level (1), at the fibular head level (2) and above the knee (3) (lower amplitudes at the proximal stimulation sites (2 and 3), especially above the fibular head (3) on the affected side (>50%). Recording is being performed here with the active electrode situated on the *extensor digitorum brevis* mucle. The temporall dispersion suggested by the decrease of amplitude (2) is also in context of the sensory-motor polyneuropathy. Note—the images are generated with Neurosoft device for EMG.

ENG revealed a reduced CMAP amplitude on the right peroneal nerve with proximal stimulations and a significant decrease in the amplitude at the most proximal stimulation site, with temporal dispersion phenomena and decreased MCV, indicating mixed axonal and demyelinating damage. The bilateral tibial nerve and bilateral superficial peroneal nerve showed normal aspects, while the left peroneal nerve exhibited a reduced CMAP amplitude along its entire length without a decrease in the MCV. For the left lower limb, the distal amplitude was 2.4 mV and the most proximal was 2.3 mV. In contrast, the right leg showed 4.5 mV distally, 1.9 mV at the fibular head, and 1.0 mV above the fibular head (Figure 7).

The EDX aspect orientated toward a PN at the fibular head and a mild radiculoplexopaty L4–L5, L5–S1.

### 3.5. Clinical Case 5

A 53-year-old female with a 13-year history of T2DM, treated with metformin and clinically diagnosed with DN, experienced a traumatic accident to her right forearm 4 years prior (no fractures or nerve damage, but with trauma to the radial fossa and recurrent inflammations). She had a chronic lesion of the palmar ligament (affecting the second to fourth fingers), with persistent numbness in the index finger and progressive joint pain and paresthesia in fingers I–IV, exacerbated by recent physical effort and repetitive hand activities while doing household chores.

**Figure 8:**
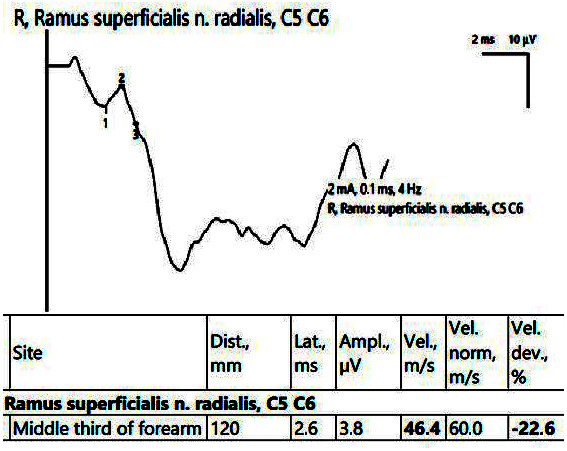
Patient 5. Superficial radial nerve evaluation (decreased sensitive conduction velocity, 46.4 m/s when stimulating in the middle 1/3rd of the forearm and collecting on the first interdigital space). Note—the images are generated with Neurosoft device for EMG.

In the affected hand, the SCV was decreased for both the median nerve in the carpal tunnel and the superficial radial nerve (Figure 8). EMG showed an active neurogenic pattern in the APB and a chronic neurogenic aspect in the first dorsal interosseous (FDI).

All the other nerve conduction parameters in the hand were within normal values (including the motor and sensory ulnar nerve). Therefore, the diagnosis was superficial radial nerve neuropathy, associated with CTS.

### 3.6. Clinical Case 6

A 43-year-old male with a 6-year history of DM, treated with metformin and SGLT2i, and associated distal DN, had a lumbar spondylodiscopathy diagnosis (confirmed by MRI, with significant disc protrusions at L4–L5 and L5–S1 levels). He presented with chronic lumbar pain radiating to the right thigh’s lateral side.

**Figure 9:**
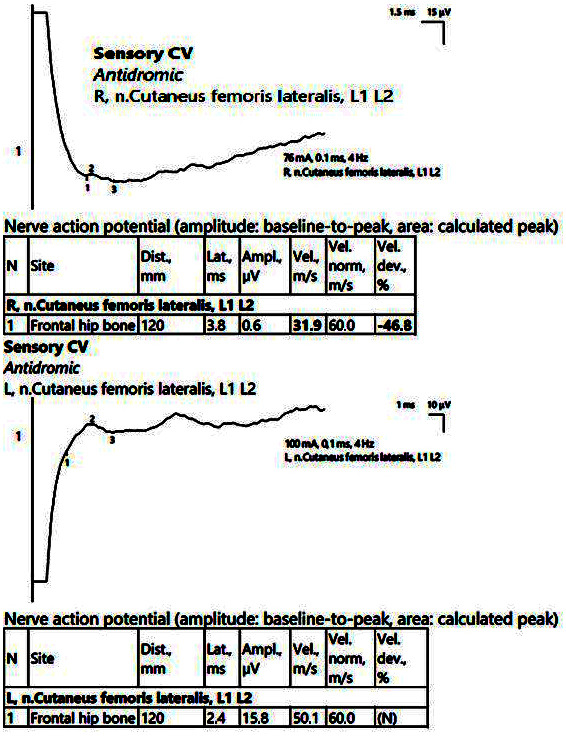
Patient 6. Right lateral femoral cutaneous nerve (decreased amplitudes on both sides, significantly reduced on the right, along with decreased velocity, 31.9 m/s). The stimulation is performed at anterior superior iliac spine level, with recording at about 12 cm on an imaginary line connecting the stimulation point with the lateral pattelar side. Note—the images are generated with Neurosoft device for EMG.

ENMG recorded low CMAP amplitudes in the bilateral peroneal nerve, with normal MCVs at this level and normal readings in the bilateral tibial nerve. However, the evaluation of the late responses/lumbar nerve roots showed asymmetries in the average latency of the F waves and asymmetries in the H reflex in the right > left peroneal nerve. There were also low SCV readings at the sural level and in the right lateral femoral cutaneous nerve (Figure 9).

EMG showed neurogenic aspects with reduced recruitment of the MUAP upon voluntary activation in the bilateral L3/L4–L5, L5–S1 investigated territories, without signs of spontaneous activity. In this case, the medical history, corroborated with the EDX findings, pointed toward a meralgia paresthetica, associated with lumbo-sacral radiculopathy.

### 3.7. Clinical Case 7

A 70-year-old male with a history of T2DM, treated with metformin and GLP-1 RAs, and DN, had experienced chronic paresthesia in the right hand, particularly in the fifth finger, for 14 years.

**Figure 10:**
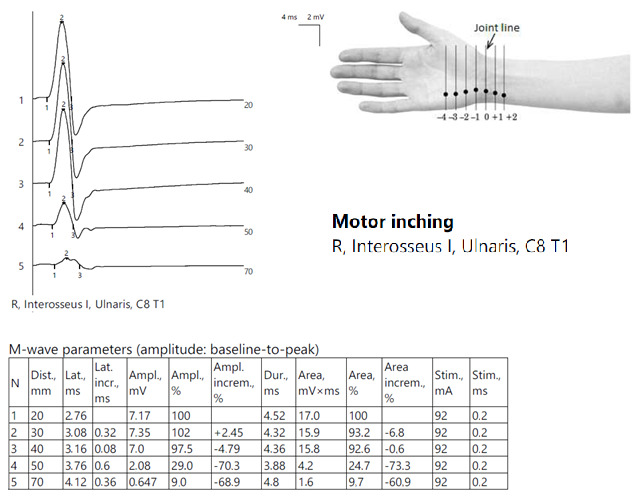
Patient 7. Motor inching across the right Guyon tunnel suggesting the decrease on amplitudes of CMAPs across the wrist’s stimulation points, with a significant decrese >50%. Note—the images are generated with Neurosoft device for EMG evaluation and it reports commas instead of dots and hyphen (-) instead of minus sign.

ENG showed globally diminished nerve conduction velocities, both motor and sensory, and a low CMAP amplitude in the right ulnar nerve at the Guyon tunnel (Figure 10).

The results orientated toward a distal UlN.

**Figure 11:**
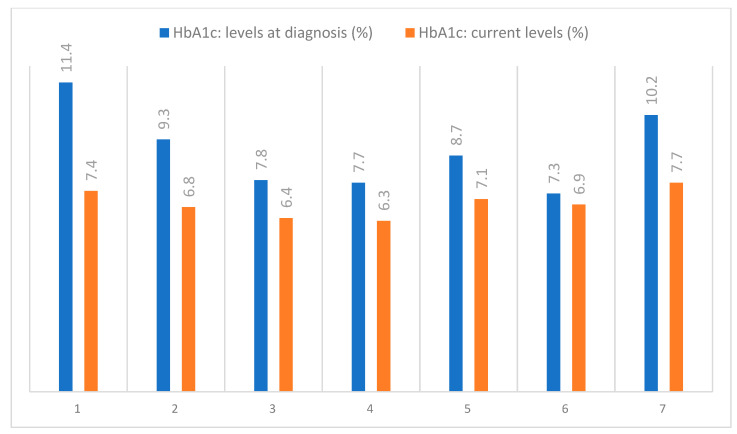
Evolution of the HbA1c levels in the examined patients.

The skin AGEs measured by means of SAF presented high values for the included patients and are shown in Table 3, alongside the HbA1c levels, which were routinely assessed, as shown in Figure 11.

## 4. Discussion

All the cases investigated had pre-existing clinical diagnoses of sensory or sensory-motor distal polyneuropathy. The presented entrapment conditions were triggered either by repetitive professional activities or by long-term repetitive compressions along the entrapment sites. We observed that all the patients exhibited elevated SAF levels above the recently reported demographic threshold of 2.35 AU for our region [27]. The higher and increased skin levels of AGEs may potentially be related to the severity of DN due to being associated with the presence of both symptoms and deficits, but no conclusions can be drawn from only the presented data due to the small number of patients observed [28]. Nevertheless, hypotheses can be generated to lay the groundwork for future research with protocols specifically designed to evaluate the true value of including SAF alongside EDX in the assessment of patients with DM.

### 4.1. Plexopathy versus Neuropathy

Radiculoplexus neuropathies in DM pose significant challenges for DDx, often causing pain in the hip or thigh. In radiculoplexopathies, particularly in long-standing T2DM, patients typically present within weeks or a few months after experiencing intense pain in the proximal thigh or pelvis. They report difficulties with general movements and exhibit substantial weakness that is disproportionate to the gradually subsiding pain [3]. The femoral and obturator nerves are frequently affected by DA, which is characterized by a noticeable wasting of the anterior and medial thigh muscles [3,4]. This context raises clinical DDx difficulties among radiculopathies related to disk hernias or even to the concept of meralgia paresthetica [29,30]. The peroneal nerve may be affected as well, orientating the diagnosis toward the notion of radiculoplexopathy, especially in the absence of a dropped foot syndrome that might suggest otherwise, as seen in the fourth patient. On the affected side, the rotulian reflex is frequently absent. In the L2–L4 distribution, there may also be relatively little sensory loss despite the noticeable discomfort, atrophy and degree of motor deficit [3,29].

Coexisting weight loss is common; however, it is fairly well understood. Patients with diabetic polyneuropathy frequently develop, simultaneously, DA; as a result, these patients may experience sensory disturbances and loss of reflexes in their distal legs [3].

While an MRI can be useful for locating a lesion physically, it only provides a snapshot in time. In contrast, EMG does not show anatomy but offers physiological information about nerve conduction and muscle-specific patterns. This information can indicate whether the condition is acute or chronic. Therefore, to confirm the diagnosis of radiculopathy, both imaging procedures (often MRI) and electrodiagnostic studies are beneficial and complementary, as demonstrated in the sixth patient [3,30].

The classic findings of DA include a clinical presentation of approximately one month of severe right gluteal and leg pain, along with specific electrophysiologic findings. Additionally, there is an absence of the right knee reflex, moderate weakness along the L2–L4 dermatomes, and no improvement with bed rest [3,4]. In our cases, we frequently considered a mixed etiology, as the patients had histories of multilevel herniated discs or radicular involvement in the context of spondylodiscopathy, conditions for which they typically undergo physiotherapy twice a year. The glycation process, accelerated in DM, leads to excessive accumulation of AGEs in connective tissues. This may contribute to the high incidence of musculoskeletal disorders and restricted joint mobility commonly observed in patients with DM [28].

### 4.2. Carpal Tunnel Syndrome in Diabetes Mellitus

Paresthesia and numbness in the thumb, index, and long fingers, as well as the radial side of the ring finger, are common signs of CTS [8,31]. Proximal irradiation and hand pain may also be experienced, which are often more noticeable at night. Patients may report hand weakness or difficulty executing fine motor skills [8,32]. Specific physical or medical issues, such as coexisting peripheral neuropathy, edema, rheumatoid arthritis, DM, pregnancy, thyroid issues, and repeated strain, increase susceptibility to CTS. However, the relationship between CTS and DM remains debatable [8,33,34]. Therefore, a comprehensive history, including work-related and environmental factors, is crucial [12]. Additionally, correlations between AGEs and CTS have been established. Glycosylation of collagen causes the collagen fibers in the transverse carpal ligament to cross-link, increasing stiffness and creating space restriction in the carpal tunnel [35]. AGEs also seem to be involved in mechanisms related to free radical activity via nicotinamide adenine dinucleotide phosphate oxidase in the development of Dupuytren’s contracture [36].

Upon physical examination, the radial dermatome examination may reveal signs of sensory impairment. It is also possible to see pinch strength weakness [37]. There may be thenar eminence wasting in severe cases of CTS [12,37]. Tests that provoke the symptoms may cause them to recur. These consist of Phalen’s test (maximum wrist flexion held for 1–2 min) and Tinel’s test (percussion of the median nerve around the wrist). It is always crucial to conduct a complete physical examination because CTS might be mistaken for other conditions [12,37,38].

To demonstrate axonal injury (fibrillation potentials or positive sharp waves) and/or reinnervation, EMG testing should be carried out. The APB muscle is accessible to investigate (second patient). Since CTS can coexist with other conditions, the testing of other muscles should be performed if spontaneous activity is observed in this particular muscle [12]. To make sure there is not a median neuropathy anywhere else along the nerve’s path, a test should be performed specifically on the more proximal median muscle. A non-median innervated C8 muscle should also be examined [3,12].

Just like in our cases, it is common that findings such as a slowing of the sensory conduction velocity in the median nerve, delays in the distal latency and low amplitudes of the CMAP, SNAP or both, orientate toward the EDX of CTS. Needle EMG in ABB can show the presence of fibrillation potential +/− positive sharp waves (most frequent signs of spontaneous pathological activity), suggesting active denervation [12].

### 4.3. From Roots to Focal Neuropathies

Assessment of clinical cases like ours suggests that in a patient with DM with the involvement of lumbar myotomes, with nerve lesions close to the roots, clinically electrophysiologic evidence of a median neuropathy may exist at the right wrist. Unlike our first patient presented, this can be totally asymptomatic. In this case scenario, the clinical diagnosis would be a DA superimposed on a broad sensory-motor peripheral neuropathy, most likely owing to DM [3]. DA, in such situations, is pathologically a radiculoplexopathy affecting the lumbar myotomes. This case also shows that it is not possible to conclusively demonstrate the plexus component by EDX in patients with both radiculopathy and peripheral neuropathy [1,3]. The question of whether a surgical intervention for a herniated disk is needed in some cases can be hard to answer. For example, if two minor central disc bulges at the L4–L5 and L5–S1 levels are found upon a lumbosacral MRI scan, but with neither the thecal sac nor the outgoing nerve roots compromised, the symptoms could not be explained by a structural defect. Patients with DM frequently have this situation when, respectively, neurologic and electrophysiologic assessments point to lumbar radiculopathy but without visible anatomical lesions. In these conditions, the diagnosis of DA should be reached carefully, taking into consideration that surgery is most likely not necessary in this situation [3,4].

There are multiple places along the ulnar nerve’s route where compression can occur. Compression most frequently happens where it is most visible, at the elbow. This typically happens when someone leans on their elbow (for example, while working at a desk) or repeatedly flexes and extends their elbow (repetitive activities) [39,40]. The injury may be caused by traction at a compression point, arthritis in the ulnar groove, ulnar collateral ligament scarring or even valgus overload. Patients usually have little and ring finger paresthesia, discomfort and numbness, which can become worse, especially when the elbow is bent [41,42]. In some scenarios, there could be associated irradiating pain all the way up the arm [40,41]. Less frequently, UlN at the wrist develops in a canal made up of the pisiform and hamate with its hook. An aponeurosis, which forms the roof of Guyon’s canal, connects these. The ulnar artery, vein, and nerve are located in this canal [3,39]. Those who exert a lot of force on their wrists, especially when extending them, such as cyclists and people assisted by the use of a cane, are susceptible to this entrapment [39].

Abduction of the fourth and fifth fingers, or Wartenberg’s sign, may manifest, particularly if the patient is asked to place their hands in the pocket of their pants. There might possibly be a Froment’s sign [3]. When a patient is instructed to hold a piece of paper between their thumb and the radial side of their second finger, this is demonstrated. The patient will substitute the adductor pollicis muscle, which is innervated by the damaged ulnar nerve, with the flexor pollicis longus muscle, which is innervated by the intact median nerve, when the examiner tries to remove the paper from the patient’s hand [3,39].

A decrease in the motor conduction velocity while performing the inching exploratory technique across the elbow, along with low CMAP and/or SNAP amplitudes (sometimes in the dorsal ulnar cutaneous branch), suggests nerve affectation, like in our two cases with proximal and distal ulnar entrapment [1,40]. Muscles innervated by the ulnar nerve may present pathological spontaneous activity during the needle EMG evaluation [39].

Both the median and the ulnar nerve may be susceptible to compression along the common entrapment sites; nevertheless, there is ongoing debate concerning the causality relation with DM [33,34,40,42].

Radial neuropathy is, at least in our experience, in the majority of cases that we come across, related to trauma, or in some cases, to the common “Saturday night palsy” etiology. Unlike motor studies that have high value in investigating the radial nerve, completed by EMG in the radial innervated muscles, merely sensorial affectations can be encountered, less frequently, in association with DM. Demyelinating lesions affecting the superficial radial sensory nerve will result in a prolonged distal delay if the lesion is located far from the source of stimulation. The site of an axonal lesion (as long as it is distal to the dorsal root ganglia) will result in a lower amplitude of the SNAP [43,44]. Radial superficial nerve is also important in patients with DM based on the sural/radial amplitude ratio of SNAP, orientated among the dying-back degeneration phenomena in the diagnosis of DN [44].

Another mononeuropathy plausible in the legs of patients with DM is the PN, which can be brought on by direct trauma, ischemia, compression or entrapment. Since the nerve is very superficial at the fibular neck (or head), this is the most likely site of compression. The patient may have a sudden foot drop of a particular attitude, although it can also happen gradually. Additionally, there can be a history of recent falls or trips. There may be paresthesia and numbness in the dorsum of the foot and lower lateral leg. Usually, there is no pain.

An extensive medical history can assist in identifying the underlying cause of the symptoms (e.g., occupational or sportive squatting, prolonged cross-legged positions). Sciatic neuropathy, lumbosacral plexopathy and lumbar radiculopathy (often L5) are compatible conditions with PN, especially in DM patients (or in our case, fourth patient) [1,45].

Assessing the peroneal nerve at the fibular neck, a decreased amplitude of CMAP and reduced velocity when stimulating above the fibular head, will be suggestive of a conduction block. The F waves can be either absent or reduced in number and the EMG findings usually orientate toward the character of denervation [46]. Within such a differential EDX, it is also useful to test muscles innervated from the tibial nerve [1]. The low amplitude of the SNAP of the superficial peroneal nerve can orientate the diagnosis when compared the sural, or in most cases of our patients, confirm a polyneuropathic distribution [45].

An entrapment neuropathy of the tibial nerve behind the medial malleolus is known as TTS. Compared to other neuropathies such as PN or CTS, the syndrome is far less common. Nevertheless, the condition may occur in patients with DM. Almost invariably, this neuropathy is unilateral. Systemic disorders, trauma, diverse lesions and/or articular pathology resulting in deformity are among the conditions that can also cause TTS and may coexist with DM. Idiopathic instances also occur occasionally [14]. Patients with TTS often report paresthesia (usually accompanied by numbness over the sole of the foot) and/or pain around the ankle, particularly medially [1]. Although cited as a neuropathic complication of DM, we did not come across novel cases of TTS reported in the mentioned period. We attribute this finding to the generally optimal HbA1c control observed in our clinical setting (mean HbA1c 7.2% across the first 2787 cases from 2024) and to the positive outcomes of the annual diabetic foot arthropathy screenings conducted in our podiatry department, following our center’s internal procedures for comprehensive foot examinations.

The optimized HbA1c levels alone do not necessarily represent a protective factor against *vasa nervorum* affectation, nor the collagen affectation. In line with experimental data showing age-related reductions in collagen solubility and increased collagen resistance to protease breakdown, aged and diabetic tissues also exhibit a distinctive yellowing of the collagen matrix [35].

It is rare for the tibial neuropathy to cause foot weakness. During a physical examination, the medial ankle tibial nerve may have a positive Tinel’s sign, as compared to individuals who suffer from a primarily sensory neuropathy [14]. These ones may experience reduced sensitivity to vibration, temperature, light touch and pinpricks. When a patient’s symptoms are mostly felt in the hands and feet, this is known as a “stocking-glove” distribution. As neuropathy is such a common feature of DM, with a long evolution, it can impact both motor and sensory fibers, as well as axon and myelin [1,14]. This leads to the question of whether needle EMG in the foot of individuals with vascular insufficiency or DN is contraindicated, which remains unanswered. Physicians who treat these patients frequently encourage them to check their feet and to stay away from mild infections that might become serious enough to endanger a limb. It is advisable to exercise extreme caution while performing needle EMG on intrinsic foot muscles in individuals with DM or substantial peripheral vascular disease, even though there are no such documented examples [3].

It most cases, we consider the ENMG necessarily only when the clinical evaluation is not enough to confirm a suspected diagnosis or the clinical signs are either asymmetrical or motor, situations that led us to this point of raising awareness throughout our selected cases regarding the possibility of focal neuropathies. This, along with the recommendation of ease of accessibility toward nerve conduction studies and EMG, especially in the current context of interdisciplinarity and the influence of diverse paraclinical data. SAF itself is not meant to be used as a screening method for DM diagnosis. Alternatively, the risk assessment of complications connected to AGE may hold value [26].

The association of focal neuropathies with the classic pattern of polyneuropathy can even worsen the prognosis for recovery, as it is known that even surgical outcomes (in CTS for example) can be negatively influenced in patients with DM [38].

### 4.4. Present and Future Interdisciplinary Perspectives and Skin Autofluorescence Considerations

Several recent articles have presented data from individuals with T2DM and have identified an important link between SAF and an elevated risk of neuropathy, as well as of several other microvascular and macrovascular DM complications. Correlations between SAF and comorbidities associated with T2DM were studied in a cohort of 825 T2DM patients by Wang et al., with their investigation revealing a significant connection between skin AGEs and diabetic sensorimotor polyneuropathy, diabetic kidney disease, diabetic retinopathy and cardiovascular disease, and, with stronger significance, with an increased number of complications [47].

Measuring skin AGEs by SAF can also screen T2DM patients for DN, suggesting clinical application. The screening was found to have moderate to poor specificity; therefore, it was suggested that SAF should be used alongside additional procedures for accuracy improvement [48]. The optimal cut-off for overall DN situates SAF ≥ 2.95 [48]. The SAF level was also capable of predicting foot ulcers in a recent study by Borderie et al. [49].

Detailing the mechanistic perspective, AGEs are produced when sugars chemically react with proteins or lipids. In patients with DM, their glucose levels are expected to be higher and have been shown to surpass those in individuals without impaired glucose metabolism. Age and prolonged exposure to hyperglycemia appear to strongly correlate with elevated SAF levels [50]. The metabolic alterations characteristic of DM accelerate the aging process, contribute to mitochondrial dysfunction, increase the accumulation of reactive oxygen species, and enhance the production of AGEs in various tissues. Another important aspect is that the axons are abundant in mitochondria; hence, they are especially susceptible to oxidative damage in diabetes mellitus. Even brief periods of hyperglycemia have been shown to promote and prolong the activation of pro-inflammatory and apoptotic mechanisms, particularly in macrophages. In 2016, Rota and Morelli explored entrapment neuropathies in patients with DM, highlighting their high prevalence regardless of the DM stage. They identified these neuropathies as neurophysiological markers of peripheral nerve dysfunction, even in the absence of other neuropathic symptoms. Their discussion revisited an older concept known as the “double crush” hypothesis to explain the underlying pathophysiological mechanisms: hyperglycemia causes harmful metabolic effects on the nerve, impacting both its structure and function—resulting in nerve edema, which constitutes the “first crush”. These changes make the nerve more vulnerable to entrapment in narrow anatomical passages, representing the “second crush”. Given the described pathophysiological processes, the elevated SAF values observed in the patients may serve as a meaningful indicator [51,52].

DN can manifest in a wide variety of signs and symptoms, in various distributions of the physiopathological mechanisms, that eventually add morbidity factors to the course of DM [53,54,55]. Patient education can help avoid or delay the occurrence of DN, in its multitude of manifestations, sometimes predictable based on EDX, which would otherwise raise the risk of neuropathic pain and trophic changes in the foot [55,56]. We also consider and promote the notion that better accessibility in relation to EDX investigations can positively impact a better diagnostic approach to the extensively studied rarer types of DN, according to the constant growing body of evidence [5,57].

The ideas promoted in terms of an interdisciplinary approach are highly relevant. Recent studies report new and interesting findings. Kalra S et al. suggest that nonspecific symptoms, such as pruritus, which are related to microvascular disease in etiopathogenic patterns, can be symptoms of DN [58]. Vitamin D remains a pertinent topic, with Pang C et al. suggesting its role in nerve conductivity and its selective relationship with DN severity among T2DM cases [59]. Other studies highlight the connections between DM and the incomplete healing of damaged tendons, as well as early parallel injury to both small and large nerve fibers in well-controlled recent-onset T2DM [60,61]. These findings underscore the need for comprehensive investigative methods, and ENMG studies fulfill this need.

The current tendency of associative investigation protocols goes along this statement, as the platelet count and plateletcrit can be associated with nerve conduction studies to characterize the unsatisfactory nerve conductivity in T2DM with DN [62]. Moreover, along with the current tendency of associating ENMG with ultrasonographic evaluation, the cross-sectional area of the tibial nerve measured by ultrasonography correlates with DN severity [63].

The advantages of EDX studies also rely on the accessibility and reasonable price of innovative point-of-care nerve conduction devices [64], or on the utility of surface EMG in rehabilitation programs, training status or measurements of aerobic exercise effect on DM with DN [65,66,67,68]. For all that, ENMG offers objective and reproducible data, useful for the assessment of nerve and muscle function impairment, association with the degree of neuropathic pain and even population studies [69,70,71].

From the classical pattern of sensory-motor distal involvement to the characterization of focal neuropathies, EMG examination can even be used to evaluate the neurogenic pattern of the external anal sphincter in some cases of T2DM [72]. Additionally, EDX not only facilitates a thorough evaluation of the degree of axonal damage due to ischemia in focal entrapment cases (such as UlN, as discussed in our cases), but it is also valuable for intraoperative perspectives in nerve decompression surgical procedures in patients with DM [73,74].

Last but not least, the utility of the motor-evoked potentials recorded by EMG surface electrodes during transcranial magnetic stimulation (TMS), along with quadriceps electrical stimulation, suggests that when compared to healthy controls, males and females with T2DM have higher knee extensor fatigability due to contractile processes [75].

Our study holds significant clinical relevance, as entrapment neuropathies are a major factor impacting quality of life, with serious implications for productivity, particularly in occupations requiring manual dexterity. This includes not only labor-intensive jobs but also office and desk-based work, especially when upper-limb pathology is involved. In cases affecting the lower limbs, specific diagnoses can guide more targeted treatments and offer valuable insights for rehabilitation specialists. This is especially important considering the substantial burden posed by lumbosacral pathology. [76].

Regardless of the small sample size, the cases presented raise awareness of frequent presentations, which should be diagnosed as soon as possible, ensuring the most effective treatment, including surgical interventions if necessary. That is, the sooner, the better, in aiming toward maintaining the patient’s independence in daily activities, as well as in professional capabilities.

The dynamics of the research in this area promote the need for associative studies; therefore, we conclude that combining the available EDX methods with skin AGE determination by SAF is both useful and accessible for dedicated medical facilities. Our results support the actual knowledge in the field, adding a new approach to the current topic.

## 5. Conclusions

Focal neuropathies represent a significant challenge in the interdisciplinary management of patients with DM. These complications can either progress silently and gradually or arise suddenly due to common entrapment risk factors. Regardless of the ongoing debate on the impact of DM on these conditions, with its specific patterns of microvascular involvement, there is a consensus that the risk is higher in this population. T2DM, in particular, predisposes patients to a higher prevalence of these complications, often triggered by common activities or positions that exacerbate the risk.

This underscores the necessity of easy access to electrodiagnostic procedures and the integration of these services into the standard activities of dedicated medical centers. The strength of this article lies in its in-depth insights into the diagnostic challenges from a clinical perspective, which can be of great value to clinicians working in multidisciplinary teams, facilitating timely referrals. ENMG is a time-consuming procedure, often taking up to one hour, and scheduling these investigations in an outpatient setting can be challenging. The determination of AGEs through SAF could help prioritize cases for EDX evaluation, although further longitudinal studies are needed to confirm this hypothesis.

A prompt and accurate diagnosis would enable quicker rehabilitation therapy or surgery, resulting in better case management and a reduction in severe complications. Despite the limitation of a small sample size, we hypothesize that integrating SAF evaluations into protocols, along with EDX for focal neuropathies, could provide significant added value, with the earlier detection of complications, particularly in silent forms of neuropathies. Our findings align with the general body of knowledge on the topic, suggesting the need for larger and more comprehensive interdisciplinary approaches.

## Figures and Tables

**Table 1:** Motor nerve conduction study values.

Nerve	Onset Latency (ms)	Amplitude (mV)	Nerve Conduction Velocity (m/s)	F Wave Latency (ms)
Median	<4.2	>4.4	>49	<31
Ulnar	<3.4	>7	>49	<32
Radial	<5.2	>4	>50	-
Peroneal (ankle site)	<5.8	>2.0	>45	<58
Tibial	<6.5	>3.0	>45	<59

**Table 2:** Sensitive nerve conduction study values.

Nerve	Onset Latency (ms)	Peak Latency (ms)	Amplitude (µV)	Nerve Conduction Velocity (m/s)
Median	<2.5	<3.5	>20	>52
Ulnar	<2.1	<3	>15	>52
Radial superficial	<1.9	<2.8	>20	>48
Sural	<4	<4.4	>6	>42
Peroneal superficial	<3.4	<3.8	>15	>42

**Table 3:** HbA1c and AGE levels of the included patients.

Patient	Current HbA1c Levels (%)	SAF (AU)
1	7.4	2.97
2	6.8	3.3
3	6.4	2.8
4	6.3	2.6
5	7.1	2.7
6	6.9	2.5
7	7.7	3.4

SAF—skin autofluorescence; AU—arbitrary units.

## Data Availability

The data supporting this study’s findings are available upon request from the authors. Please note that the data are also being utilized in an ongoing study investigating the benefits of including skin autofluorescence in predicting neuropathy in patients with focal neuropathies.
